# Tarsal tunnel syndrome secondary to osteochondroma of the calcaneus: a case report

**DOI:** 10.1186/s12891-020-03530-9

**Published:** 2020-07-25

**Authors:** Sung Hun Won, Jahyung Kim, Tae-Hong Min, Dong-Il Chun, Young Yi, Sang Hak Han, Jaeho Cho

**Affiliations:** 1 Department of Orthopaedic Surgery, Bone & Joint center, Soonchunhyang University Seoul Hospital, Seoul, Republic of Korea; 2 Department of Orthopaedic Surgery, Seoul Hospital, Soonchunhyang University Seoul Hospital, Seoul, Republic of Korea; 3 Department of Orthopaedic Surgery, Seoul Paik Hospital, Inje University, Seoul, Republic of Korea; 4 Department of Pathology, Chuncheon Sacred Heart Hospital, Hallym University, Chuncheon, Republic of Korea; 5 Department of Orthopaedic Surgery, Chuncheon Sacred Heart Hospital, Hallym University, 77, Sakju-ro, Chuncheon-si, Gangwon-do 200-704 Republic of Korea

**Keywords:** Osteochondroma, Tarsal tunnel syndrome, Calcaneus

## Abstract

**Background:**

Tarsal tunnel syndrome is an entrapment neuropathy that can be provoked by either intrinsic or extrinsic factors that compresses the posterior tibial nerve beneath the flexor retinaculum. Osteochondroma, the most common benign bone tumor, seldom occur in foot or ankle. This is a rare case of tarsal tunnel syndrome secondary to osteochondroma of the sustentaculum tali successfully treated with open surgical excision.

**Case presentation:**

A 15-year-old male presented with the main complaint of burning pain and paresthesia on the medial plantar aspect of the forefoot to the middle foot region. Hard mass-like lesion was palpated on the posteroinferior aspect of the medial malleolus. On the radiological examination, 2.5 × 1 cm sized bony protuberance was found below the sustentaculum tali. Surgical decompression of the posterior tibial nerve was performed by complete excision of the bony mass connected to the sustentaculum tali. The excised mass was diagnosed to be osteochondroma on the histologic examination. After surgery, the pain was relieved immediately and hypoesthesia disappeared 3 months postoperatively. Physical examination and radiographic examination at 2-year follow up revealed that tarsal tunnel was completely decompressed without any evidence of complication or recurrence.

**Conclusions:**

As for tarsal tunnel syndrome secondary to the identifiable space occupying structure with a distinct neurologic symptom, we suggest complete surgical excision of the causative structure in an effort to effectively relieve symptoms and prevent recurrence.

## Background

Tarsal tunnel syndrome is an entrapment neuropathy of the posterior tibial nerve or one of its branches within the tarsal tunnel on the medial aspect of the ankle [1]. Symptoms of burning, tingling, and pain along the foot plantar region can be triggered by either intrinsic or extrinsic factors that compress the posterior tibial nerve beneath the flexor retinaculum [2]. Among the rest, a bony structure protuberated within the tarsal tunnel including talocalcaneal coalition, talar or calcaneal fracture, nonunion, or os sustentaculum tali have been identified as one of the significant factors to tarsal tunnel syndrome due to its firm mass effect [1, 3–7]. Osteochondromas, the most common benign bone tumor also characterized by a stalk or a flat protuberance emerging from the surface of the bone, however, rarely occur in tarsal bones [8–10]. Here, we report a patient who presented with tarsal tunnel syndrome secondary to osteochondroma of the sustentaculum tali and was successfully treated through surgical decompression.

## Case presentation

### Preoperative evaluation

A 15-year-old male presented with insidious onset of pain and a palpable mass on the left foot for 1 year. The burning pain was combined with paresthesia on the medial plantar aspect of the forefoot to the middle foot region, along the innervation of the medial plantar nerve. The symptoms were aggravated upon walking or prolonged standing and were relieved at rest. No previous trauma or surgical history was reported. On physical examination, there was a fixed, hard mass-like lesion palpated on the posteroinferior aspect of the medial malleolus. The range of motion of the lower extremity including subtalar motion was normal and no evidence of hindfoot malalignment was detected. Light touch and pin prick were performed and the patient complained of hypoesthesia of 80% the normal contralateral leg along the medial plantar foot area. A positive Tinel’s sign was detected.

**Fig. 1:**
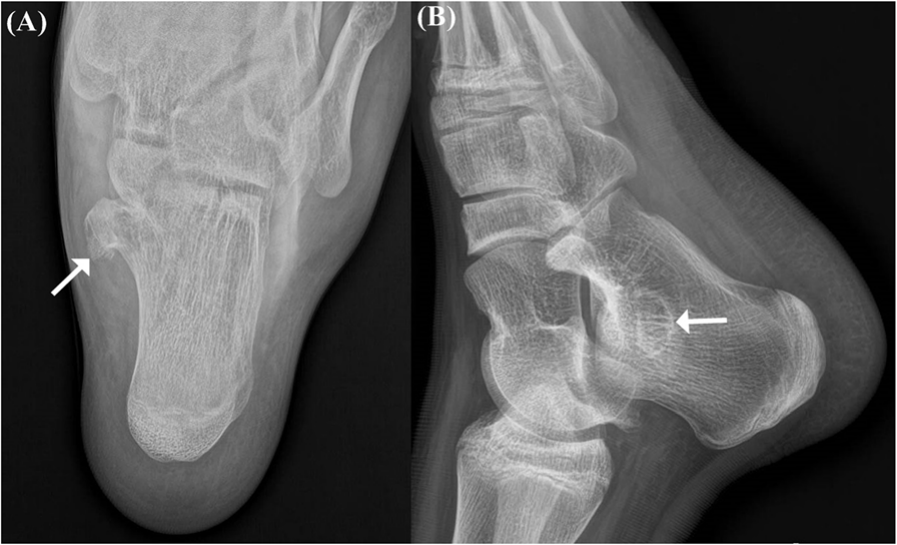
Preoperative axial (**a**) and lateral (**b**) plain radiographs of the calcaneus. A large peduculated mass is located posteroinferomedial to the sustentaculum tali. Corticomedullary continuity is observed between the mass and the sustentaculum tali (arrows)

**Fig. 2:**
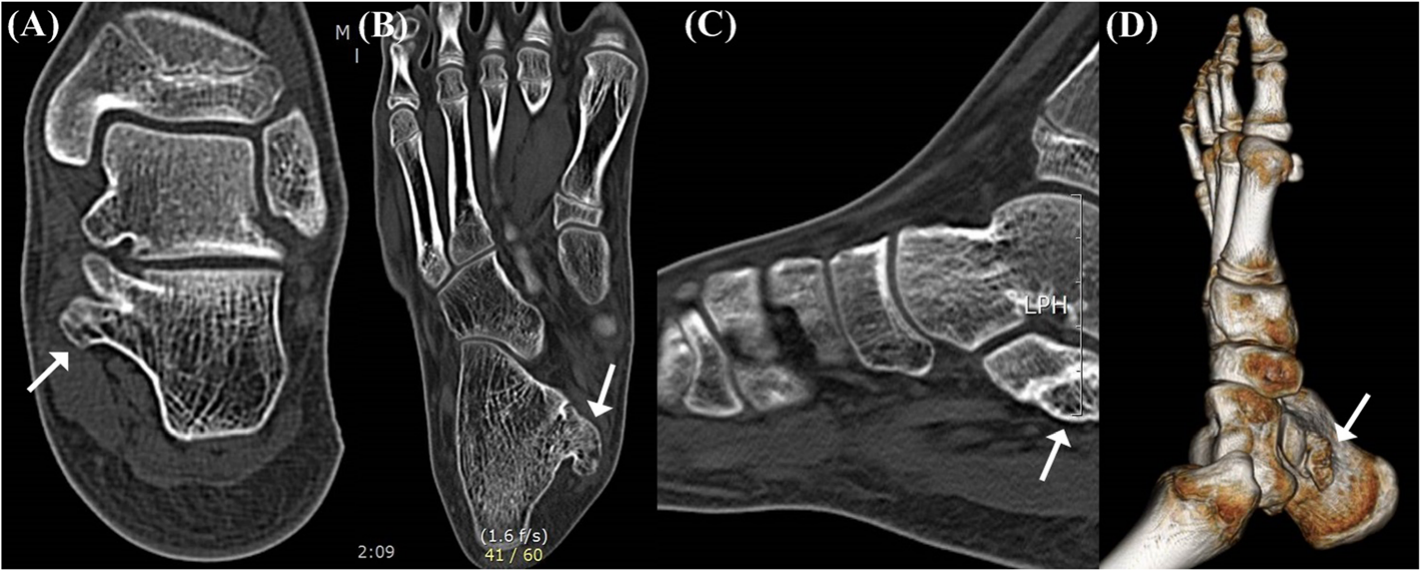
Preoperative CT scans of the left foot. Coronal (**a**), axial (**b**), sagittal (**c**), and three dimensional reconstructed (**d**) views show 2.5 × 1 cm sized bony protuberance below the sustentaculum tali, demonstrating an apparent corticomedullary continuity with the underlying bone (arrows). Neither talocalcaneal coalition nor fracture within the bony protuberance is detected

**Fig. 3:**
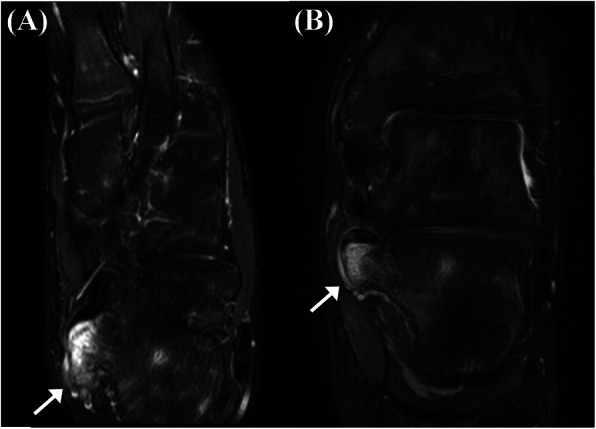
Preoperative MRI scans show the high T2 signal intensity mass extended from the sustentaculum tali occupying the tarsal tunnel (**a**, **b**). The fatty bone marrow is seen on the central portion of the mass, continuously with the marrow cavity of the underlying bone, surrounded by thin cartilaginous cap (arrows)

The plain radiographs of both axial and lateral views of the calcaneus showed a large pedunculated mass located posteroinferomedial to the sustentaculum tali. In addition, corticomedullary continuity between the sustentaculum tali and the mass could be detected (Fig. 1). No other visible pedunculated lesion could be detected on the whole body x-ray. To clarify the lesion, a computed tomography (CT) was performed, which showed a 2.5 × 1 cm sized bony protuberance below the sustentaculum tali, demonstrating an apparent corticomedullary continuity with the underlying bone. Neither the talocalcaneal coalition nor fracture within the bony protuberance was detected (Fig. 2). Magnetic Resonance Image (MRI) scans confirmed the high T2 signal intensity mass extended from the sustentaculum tali, occupying the tarsal tunnel. The fatty bone marrow was observed on the central portion of the mass, continuously with the marrow cavity of the underlying bone, surrounded by thin cartilaginous cap (Fig. 3). The Foot and Ankle Outcome Score (FAOS) was 81.11 points [11]. Electromyography was not performed due to typical signs and symptoms of tarsal tunnel syndrome and surgical intervention was indicated due to presence of tunnel occupying lesion.

### Surgical procedure

**Fig. 4:**
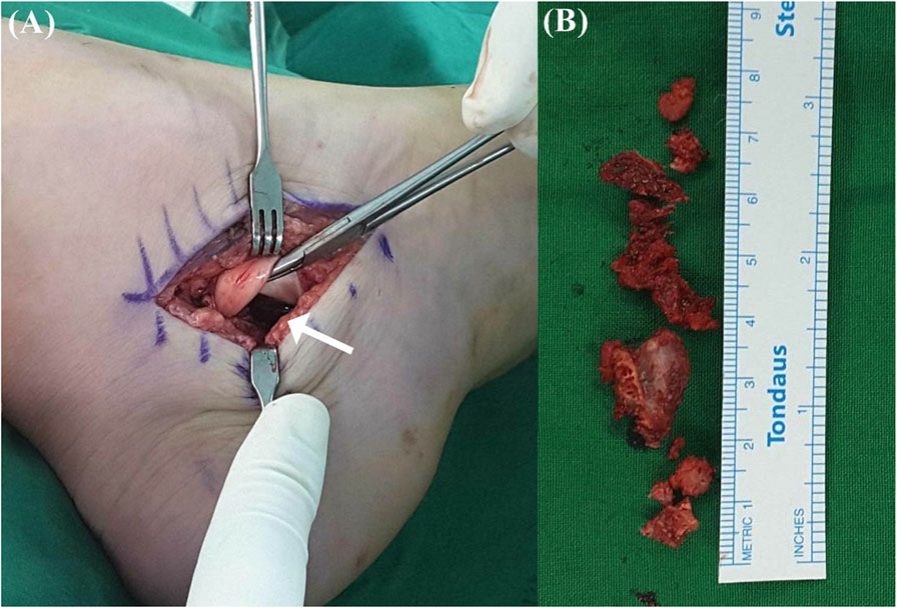
Intraoperative photographs. **a** Sufficient blank space (arrow) under the retracted tendon, occupied after complete excision of the osteochondroma. **b** Bony mass meticulously excised from sustentaculum tali using the osteotomes and rongeur

Surgical excision of the bony mass was performed under the general anesthesia. The flexor retinaculum was released and 2.5 × 1 cm sized bony mass was found on the posteroinferior border of the sustentaculum tali, compressing the posterior tibial nerve underneath. No talocalcaneal coalition bar was detected. The bony lesion was completely excised from the sustentaculum tali using the osteotomes and rongeur until no remaining protuberance was seen within the tunnel, and the tension on the nerve was eventually released (Fig. 4). Excised bony mass was sent to the pathology department for histologic examination.

### Postoperative progression

**Fig. 5:**
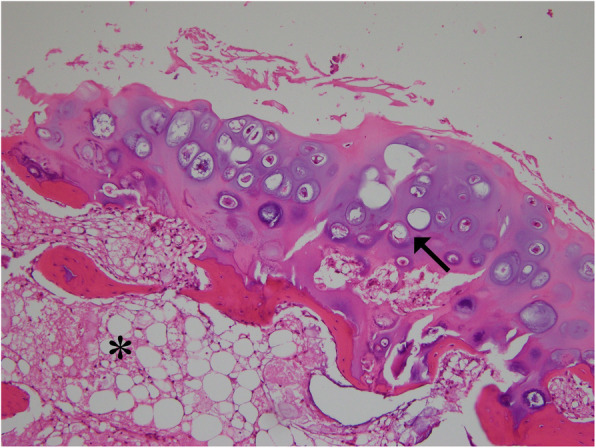
The microscopic appearance of an osteochondroma displays the benign cartilaginous cap with columns of chondrocytes (arrow) at the top and the bony cortex with marrow space at the bottom (asterisk). (Haematoxylin and eosin, × 100)

The histologic sample was confirmed to be osteochondroma (Fig. 5). A below-knee splint was applied for 2 weeks and the patient was allowed to fully bear weight and return to the regular activity after the stitches on the wound were removed. The patient’s pain was relieved immediately after surgery and the hypoesthesia disappeared 3 months postoperatively. No complications or recurrence of symptoms were detected at the 2-year follow-up and the FAOS improved to 100 points.

## Discussion and conclusions

Osteochondroma is the most common benign bone tumor accounting for more than 30% of all the benign bone tumors [12]. It is defined as a cartilage capped bony projection arising on the external surface of bone containing a marrow cavity that is continuous with that of the underlying bone [8]. It tends to be found in adolescent or children and no predilection among the gender exists. Most of the lesion occur as solitary, nonhereditary lesion, which is usually located at the metaphysis of the long bones, especially near the knee joint [8, 10]. Osteochondroma is seldom found in foot or ankle, excluding the case of Multiple Hereditary Exostoses [13].

In terms of differential diagnosis of the osteochondroma near the ankle, Dysplasia epiphysealis hemimelica (DEH) could also be taken into account because it is mostly affected in ankle and knee [14]. DEH is a rare developmental disorder defined as asymmetric osteochondral overgrowth of the cartilage of epiphysis or epiphyseal equivalent [15]. It is characterized as an intra-articular, irregular bony lesion with osseous continuity to the underlying bone arising from multiple distinct ossification centers. Although the histopathologic study confirmed the lesion of this case report as osteochondroma, it is known to be difficult to discriminate osteochondroma from DEH both clinically and pathologically [16, 17]. In fact, calcaneus is an apophyseal equivalent and DEH occurs predominantly on male in developmental ages, just as the patient in this case report [18]. Despite the definite diagnostic value of the molecular assays, these are costly and radiological findings are considered to be important in discriminating these two lesions [19]. In this case, the lesion was located under the sustentaculum tali, which could be considered extra-articular. In addition, the cortical and medullary continuity between the lesion and the sustentaculum tali was apparent compared with that of DEH [15]. Lastly, no additional lesion could be detected in other ossification centers on the whole-body x-ray, excluding the generalized form of DEH. For these reasons, we thought it would be appropriate to regard the lesion as the osteochondroma.

Patients with osteochondroma usually do not have symptoms, excluding the discomfort caused by mechanical irritation of the mass. However, if osteochondroma occur near the neurovascular structure, mostly popliteal nerve and artery around knee, numbness, weakness, loss of pulse, and color change can be presented on the affected limb [20]. Patient in our case was put into a similar situation by the osteochondroma originated from the rarely affected structure, the sustentaculum tali. As a result, tarsal tunnel syndrome was provoked.

Surgical intervention for tarsal tunnel syndrome is indicated among symptomatic patients who do not respond to non-operative treatment or those who have space-occupying lesion within the tarsal tunnel [2, 21]. Besides, a positive Tine’s sign is considered one of the most predictable indicators to the satisfactory outcome after the surgery [1]. The standard surgical approach to release the compressed posterior tibial nerve in the tarsal tunnel syndrome is known to be open tarsal tunnel decompression [1]. Our surgical success in this case seems to have been derived from radical excision of the bony mass because incomplete removal of the osteochondroma and inadequate tarsal tunnel release are together related to the recurrence and residual symptoms [12, 22].

One previous case report has demonstrated a similar case of tarsal tunnel syndrome secondary to osteochondroma of the sustentaculum tali [23]. However different from our case, the patient had undergone tarsal tunnel release prior to presentation and the talocalcaneal coalition was detected along with the osteochondroma. Scar tissues resulted from the previous operation and the mass effect of the talocacaneal coalition may have affected the compression of the posterior tibial nerve in combination so the talocalcaneal bar resection was performed additionally. In this study we were able to effectively decompress the posterior tibial nerve by solitarily excising the osteochondroma followed by immediately diminished the symptoms.

In conclusion, we encountered a rare case of tarsal tunnel syndrome secondary to osteochondroma of the sustentaculum tali in pediatric patient. If the space occupying structure within the tunnel is identified with a distinct neurologic symptom, complete surgical excision of the causative structure can bring out significant improvement of preexisted symptoms without recurrence.

## Data Availability

Data sharing is not applicable to this article as no datasets were generated or analysed during the current study.

## Abbreviations

CT: Computed Tomography
FAOS: Foot and Ankle Outcome Score
MRI: Magnetic Resonance Image
